# The Impact That Different Types of Organizational Cultures Have on the Adjustment of Self-Initiated Expatriates

**DOI:** 10.3389/fpsyg.2021.804947

**Published:** 2022-01-13

**Authors:** Vilmantė Kumpikaitė-Valiūnienė, Ineta Žičkutė, Irma Banevičienė, Junhong Gao, Denisse Torres

**Affiliations:** ^1^School of Economics and Business, Kaunas University of Technology, Kaunas, Lithuania; ^2^College of Economics and Management, Shandong University of Science and Technology, Qingdao, China

**Keywords:** organizational culture, culture types, self-initiated expatriates, adjustment, work adjustment, non-work adjustment

## Abstract

This paper investigates the adjustment of self-initiated expatriates, with a particular emphasis on organizational culture. One hundred and twenty-five self-initiated expatriates around the globe participated in the online survey. We examined the impact that organizational culture has on self-initiated expatriate work and non-work-related adjustment using multiple linear regression analysis. Four types of organizational culture (clan, adhocracy, market, and hierarchy) were explored. The results revealed that Clan culture has a positive effect on the work and non-work-related adjustment of self-initiated expatriates.

## Introduction

Economic globalization requires an increasing number of people to work abroad for short or long periods of time (Cappellen and Janssens, 2010). This includes expatriates (Al Ariss and Özbilgin, 2010; Andresen et al., 2014; Cerdin and Le Pargneux, 2014) and migrants (Guo and Al Ariss, 2015; Hajro et al., 2019) who move abroad for work-related reasons. All of these individuals need to adjust to their new countries and organizations (Black et al., 1991; Haslberger et al., 2014). The multidimensionality of this adjustment process has been discussed in both acculturation and expatriation literature. Researchers who study the adjustment process that expatriates undertake focus on different aspects of their experience, such as adjustment models and types of adjustment (Lysgaard, 1955; Black et al., 1991; Waxin, 2004; Liu and Lee, 2008; Haslberger et al., 2014), adjustment processes (Shaffer et al., 1999; Chen et al., 2002; Rosenbusch and Cseh, 2012), cultural shock (Oberg, 1960; Sims and Schraeder, 2004; Qin and Baruch, 2010; Rajasekar and Renand, 2013), or factors that influence adjustment (Black, 1990; Black and Gregersen, 1991; Aycan, 1997; Hechanova et al., 2003; Mezias and Scandura, 2005; Selmer and Lauring, 2009; Barner-Rasmussen et al., 2014; Feitosa et al., 2014). Moreover, most of the research on expatriate adjustment focuses on individual factors that predict adjustment and performance without paying adequate attention to organizational antecedents (e.g., Black, 1990; Barner-Rasmussen et al., 2014).

The type of organizational culture (Pinto et al., 2011) and cultural novelty (Black and Gregersen, 1991) have been noted to be organizational antecedents of adjustment. Even though some studies identified a negative association between culture novelty and adjustment (e.g., Black and Stephens, 1989; Shaffer et al., 1999; Bhaskar-Shrinivas et al., 2005; Pinto et al., 2011), less attention has been paid to the influence that a given type of organizational culture has on adjustment. Therefore, the lack of research on organizational factors, particularly on the influence of organizational culture type, is apparent. Cultural novelty is defined as a person’s perception of how different from each other are home and host country (Black and Stephens, 1989). An organization’s cultural novelty is usually analyzed by comparing the organizational cultures of an assigned expatriate’s home and host countries; however, there is currently an increasing demand for self-initiated expatriates and their growing mobility (Fee and Gray, 2020). Two types of expatriates are most commonly analyzed in the scientific literature: assigned expatriates (AEs) and self-initiated expatriates (SIEs). AEs are described as employees (mostly professionals or managers) who are sent by their employer to a foreign subsidiary (Suutari and Brewster, 2000; Andresen et al., 2014; Przytuła, 2015; McNulty and Brewster, 2017). In comparison with AEs, SIEs could be described as individuals who are not sent abroad by their organization but instead decide to look for international work experience on their own (Suutari and Brewster, 2000; Meuer et al., 2019; Andresen et al., 2020). By their initiative, SIEs join a new organization, which usually has nothing in common with the previous company that they worked for in their home country.

Most studies on expatriate adjustment focus on AEs (e.g., Aycan, 1997; Andreason, 2003; Pinto et al., 2012). The same could be said about research that centers on organizational culture type and adjustment (e.g., Black et al., 1991; Pinto et al., 2011, 2012). This means that current research overlooks the potential influence of organizational culture type on the adjustment of SIEs, which is a gap that this study aims to fill.

Our paper is organized in the following way. First, our theoretical framework is presented. This initial section begins with a discussion about the adjustment of expatriates and is followed by an introduction to the different organizational culture typologies and the links between organizational culture types and expatriate adjustment. The research instrument, research sample, and procedure are explained in the methodology section, as are the results of the quantitative research. A final discussion and an overview of conclusions, limitations, future research directions, and practical implications appear at the end of the paper.

## Theoretical Framework

### Self-Initiated Expatriate Adjustment

The intercultural adjustment of expatriates is defined as a ratio of human psychological comfort and knowledge of a foreign culture (Waxin and Panaccio, 2005). Unlike AEs, SIEs are not sent abroad by a company and thus do not undergo any preparation for their adjustment to a new organization abroad. Therefore, in their case, anticipatory adjustment, which is utilized in the case of AEs (e.g., Black and Gregersen, 1991), is not formalized, so only in-country adjustment should be highlighted.

According to the most popular classification system, which was created by Black (1990), adjustment after arriving in the host country could be divided into (1) work adjustment, which refers to the comfort associated with the new job requirements abroad; (2) interaction adjustment, which refers to the adjustment associated with the socialization that takes place between the expatriate and their host country’s nationals, both at work and beyond; and (3) general or cultural adjustment, which refers to the foreign culture and living conditions abroad.

Some additional classifications of expatriate adjustment could be noted. While exploring individual- and organizational-level predictors for expatriate adjustment, Aycan (1997) analyzed the relationship between general adjustment and work adjustment and did not include interaction adjustment. Similarly, in presenting a framework of international adjustment, Haslberger et al. (2014) highlighted work adjustment and non-work adjustment dimensions. The authors consider interaction adjustment to be a subset of both work and general (non-work) adjustment, explaining that interaction takes place in the work environment (e.g., with other employees or customers) and in general non-work environments (e.g., with people in public or neighbors) (Haslberger et al., 2014, p. 342). We agree with this type of approach and thus focus on work and general non-work adjustment in our study.

Non-work adjustment is related to the environment and factors outside of an organization. Country culture novelty, spouse or family adjustment, and individual features, such as previous international experience, host-language skills, and personal characteristics, could be highlighted as non-work factors that influence expatriate adjustment (Andreason, 2003; Bhaskar-Shrinivas et al., 2005; Pinto et al., 2012).

The work adjustment of expatriates is characterized by both good performance and positive attitudes toward their new work role (Aycan, 1997). While analyzing antecedents of adjustment, Black and Gregersen (1991) highlighted the role of work-related factors, such as job factors and organizational factors. Job factors include role clarity, role discretion, role novelty, and role conflict (Stroh et al., 1994; Pinto et al., 2011). Organizational factors include organizational cultural novelty and social support (Andreason, 2003). As studies on organizational factors’ influence on work adjustment concentrated on AEs; the direct application of the terms is not suited for analysis of SIEs work adjustment. Social support for AEs consists of support from the home office, support from co-workers in the host country’s subsidiary, and as SIEs had no home office to expect support from, we will not expand on the types of support SIEs receives. Organizational cultural novelty in previous studies concentrated on differences between the cultures at overseas subsidiary and at the home office where AE was sent from Black et al. (1991). In association with SIEs, the organizational cultural novelty would compare differences between the previous workplace and the new workplace taking different countries out of the context. Therefore, SIEs are more similar to national employees entering a new organization, and so we propose that organizational culture type rather than organization culture novelty could have an impact on SIEs work adjustment.

### Links Between Organizational Culture Typologies and Expatriate Adjustment

Organizational culture could be defined as “shared values and basic assumptions that explain why organizations do what they do and focus on what they focus on” (Schneider et al., 2017, p. 468). These shared values are grounded in the history of an organization and are valid because they have been proven to be effective (Schein, 2010). New members in an organization should adjust to the values that are a part of the organizational culture. If the values are similar to the individual values of the acceptance of these values and transition into the organization become easier. How well a new employee fits into an organization could be described using the person-organization (P-O) fit theory. P-O fit theory is defined as “the compatibility between people and organizations which occurs when: (a) at least one entity provides what the other needs, or (b) they share similar fundamental characteristics, or (c) both” (Kristof, 1996, p. 45). Fit in managerial literature is identified by how similar an individual’s characteristics (personality, values, goals, and attitudes) are to an organization’s characteristics (organizational culture, values, goals, and norms) (Kristof, 1996; Lee and Wu, 2011). O’Reilly et al. (1991) revealed that organizational culture helps determine a person’s “fit” within a particular organization because “fit” includes feeling comfortable with the culture. Hofstede (1993) research highlighted that individual values and organizational practices need to be integrated to increase the degree of P-O fit. Based on these findings, we propose that a particular type of organizational culture increases P-O fit and has a positive impact on the adjustment of expatriates.

Studies that focus on expatriate adjustment also highlight the importance of organizational culture (e.g., Black, 1988; Black et al., 1991; Pinto et al., 2011, 2012). The similarity between a parent organization’s culture and the host’s organization’s culture also positively influences expatriate adjustment (Liu and Shaffer, 2005). As noted before, though, these types of studies mostly focus on AEs and proposed features, such as organizational culture novelty, social support, and logistical help (Black et al., 1991), which do not apply to SIEs because they are not connected to a parent organization; however, based on previous studies on expatriation (Black et al., 1991; Pinto et al., 2011, 2012), organizational culture could foster the work and non-work adjustment of expatriates, and one of the traditional typologies of organizational culture could measure it.

There are many typologies of organizational culture (e.g., Quinn and McGrath, 1985; Cameron et al., 1991; Trompenaars, 1994; Schneider et al., 1995; Goffee and Jones, 1988; Cameron and Quinn, 1999), but the majority of classifications are similar. However, probably the most popular classification is by dividing culture to *Clan, Adhocracy, Market*, and *Hierarchy* (e.g., Quinn and Rohrbaugh, 1983; Cameron et al., 1991; Cameron and Quinn, 1999).

The Clan and Adhocracy types could be described as dynamic and flexible cultures; however, the Clan culture is focused on employee involvement and friendly communication in the organization (Hartnell et al., 2011). Clan culture is characterized by high sociability, friendly relationships, and informality between group members. It is based on high communication and the values of loyalty and interpersonal connections, such as family or teamwork (Barner-Rasmussen et al., 2014). Furthermore, Clan culture is marked by employee commitment and concern and trust in people. We assume that this kind of employee-friendly culture supports new employees and fosters informal interactions that allow expatriates to gather more information related to non-work-related factors, which could be useful for their everyday lives.

Adhocracy culture is oriented around creativity and growth, risk-taking, and autonomy (Hartnell et al., 2011). Focus on community impact and a need for a high degree of flexibility and individuality are the key values for Adhocracy culture. The glue that holds the organization together is a commitment to creativity and innovation (Boggs, 2004). Furthermore, the culture encourages individual initiative and the freedom of employees. We assume that freedom and initiative could help expatriates better adjust and fit the organization. In addition, Adhocracy organization has external focus and differentiation (Cameron and Quinn, 1999), which, as we propose, could foster better non-work adjustment outside the organization.

While exploring the impact of organizational culture types on marketing professionals in the United States, Lund (2003) pointed out that organizational cultures that are guided by fraternal relationships, good mentors, and respect for the individual foster a higher level of adjustment. Focusing on the organizational culture typology created by Cameron et al. (1991), Lund (2003) highlighted that the Clan and Adhocracy types, which emphasize spontaneity and flexibility, encourage better adjustment. Therefore, we propose:

*H1. Clan culture is positively associated with (a) expatriate work adjustment and (b) expatriate non-work adjustment.*

*H2. The* Adhocracy *culture is positively associated with (a) expatriate work adjustment and (b) expatriate non-work adjustment.*

The Market culture is centered on competitiveness and achievement among employees (Cameron and Quinn, 1999). The main priority in this type of organization is competition and getting the job done. Its efficiency is measured in terms of achieving the objective. Stability, control, external focus, and differentiation are the main pillars of such an organization (Cameron and Quinn, 1999). The leader is a strict supervisor and demands excellence from the employees. This could influence the stress of employees. Looking at the provided characteristics of the Market culture, we propose that they do not promote the adjustment of expatriates and their fit into the organization. Therefore, the following hypothesis can be formulated:

*H3. Market culture is negatively associated with (a) expatriate work adjustment and (b) expatriate non-work adjustment.*

The Hierarchy type emphasizes clearly defined roles for employees and highly formalized structures (Cameron and Quinn, 2006). The key value is control in the hierarchy organization. In addition to a high level of control, this culture could be described by bureaucracy, rules, and regulations and is characterized by the security of employment, conformity, predictability, and stability in relationships. Hierarchy culture focuses on internal maintenance and integration and a need for stability (Cameron and Quinn, 1999). The leaders pride themselves on being good coordinators and organizers who are efficiency-minded (Boggs, 2004). This type of culture does not develop deeper relationships among employees and is not friendly to new employees. We propose that a high level of control with plenty of rules and procedures, lack of flexibility and inner orientation do not help new expatriates to adjust either to the organization or outside it. Therefore, we formulate this final hypothesis:

*H4. The Hierarchical culture type is negatively associated with (a) expatriate work adjustment and (b) expatriate non-work adjustment.*

**FIGURE 1:**
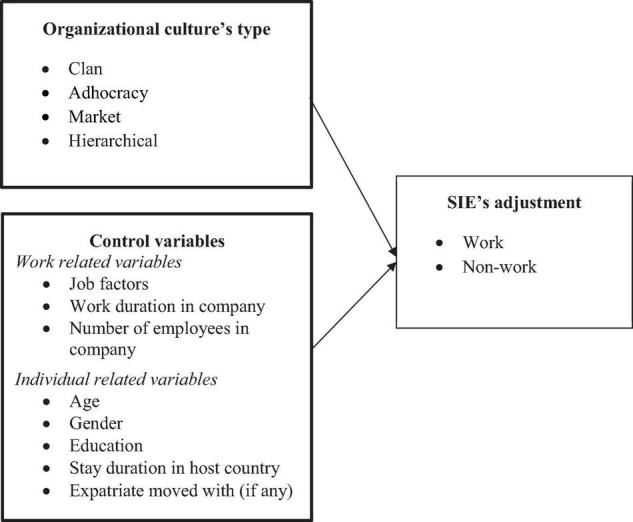
Research model.

Based on the expatriate adjustment models of Quinn and McGrath’s (1985) and Black et al. (1991) and Haslberger et al. (2014) organizational culture typology, our research model is presented in Figure 1.

## Methodology

### Measures

Measures for the study were selected based on the expatriation model of Haslberger et al. (2014), the expatriate adjustment questionnaire of Black et al. (1991), and Cameron and Quinn (1999) organizational culture typology.

#### Dependent Variables

Work and non-work adjustment were evaluated according to the expatriate adjustment model of Black et al. (1991). The questionnaire was adapted for SIEs, and all questions related to AEs were excluded (e.g., *“I would like to stay longer than assigned”*). *Work adjustment* was determined by four statements, such as *“The work in this organization so far has been successful”* or *“I feel adjusted to my organization*.” The Cronbach Alpha of the scale was 0.886. Five statements, including reversed (R) items, measured *non-work adjustment* (e.g., *“The move so far has been successful*,*” “It is difficult for me to adjust (R)*,*” “I feel adjusted to this country”*). The Cronbach Alpha of the scale was 0.887.

#### Independent Variables

We used Cameron and Quinn (1999) organizational culture typology (i.e., Clan, Adhocracy, Market, and Hierarchical). Organization culture typology was assigned using an organizational culture assessment instrument (David et al., 2018). Each organizational culture typology was evaluated by providing six statements for every culture. For instance, Clan culture examples consisted of statements such as *“The organization is a very personal place. It is like an extended family. People seem to share a lot of themselves”* and *“The management style in the organization is characterized by teamwork, consensus, and participation*.” The Cronbach Alpha of the scale was 0.814. Adhocracy culture examples consisted of statements such as “*The organization is a very dynamic entrepreneurial place. People are willing to stick their necks out and take risks”* and *“The organization emphasizes acquiring new resources and creating new challenges. Trying new things and prospecting for opportunities are valued*.” The Cronbach Alpha of the scale was 0.818. Examples that fell under the Market culture classification included statements such as *“The organization is very results-oriented. A major concern is getting the job done. People are very competitive and achievement oriented”* and *“The organization defines success on the basis of winning in the marketplace and outpacing the competition. Competitive market leadership is key*.” The Cronbach Alpha of the scale was 0.744. Finally, Hierarchical culture was described through statements such as *“The organization is a very controlled and structured place. Formal procedures generally govern what people do”* and *“The organization defines success on the basis of efficiency. Dependable delivery, smooth scheduling and low-cost production are critical*.” The Cronbach Alpha of the scale was 0.803.

#### Controlling Variables

Additional individual and work-related variables were incorporated into the questionnaire. *Individual related variables* consisted of age, gender, education, stay duration in the host country, the people who an expatriate moved with (if any), and citizenship status in the host country. Meanwhile, *work-related variables* included job factors, work duration in the company, and the number of employees in the company. Job factors, such as role clarity and freedom of actions, were measured according to the Black et al. (1991) questionnaire and evaluated using five statements, including reversed item: *“My job responsibilities are clearly defined,” “It is difficult to figure out my work role (R),” “I am given the freedom to define my work role*.” The Cronbach Alpha of the scale was 0.642.

A five-point Likert scale from 1 (strongly disagree) to 5 (strongly agree) was used for the measurement of work and non-work adjustment, organizational culture, and job factors.

### Sample and Procedure

Data for this empirical study have been collected from expatriates around the world by distributing the surveymonekey.com online survey through the system portal surveymonekey.com. We shared the questionnaire on social media, especially in expatriate groups with members who lived across the globe. We also distributed the questionnaire to contacts around the world and shared it with expatriates on the www.internations.org contact page through direct messages. The questionnaire was written in English.

We collected data in March-May and September-December of 2019. We found many uncompleted questionnaires after the first phase, and therefore we shared our questionnaire link again after summer. In total, we received questionnaires from 259 respondents, of whom 43.2% of the questionnaires were incomplete. Thus, due to insufficient data, incomplete questionnaires were not further analyzed. The focus of the study was SIEs. The status of SIE was tested by the question “By whose initiative did you move abroad?” and all answers if respondents were sent by the organization were excluded from the final analysis. The final sample used for the analysis consisted of 125 SIEs working all over the world. All analysis was carried out using SPSS, version 27.

## Results

### The Descriptive Statistics

The respondent group was very diverse. The total sample includes 125 respondents from 41 different countries working in 33 host countries. The descriptive statistics of the sample are presented in Table 1.

**TABLE 1:** Descriptive statistics.

		*N*	*%*	Mean	*SD*
**Dependent variables**					
Work adjustment		125		3.94	0.85
Non-work adjustment		125		3.97	0.80
**Individual related variables**					
Age*				35.14	9.87
Gender	Female	73	58.4		
	Male	52	41.6		
Education	High school degree	14	11.2		
	BA, MA, PhD	111	88.8		
Stay duration in host country	Up to 6 months	15	12.0		
	6–11 months	10	8.0		
	1–2 years	25	20.0		
	3–5 years	21	16.8		
	More than 5 years	54	43.2		
Expatriate moved with (if any)*	Alone	66	52.8		
	Spouse	30	24.0		
	All family	28	22.4		
**Work related variables**					
Job factors		125		3.49	0.71
Work in company duration	Up to 6 months	32	25.6		
	6–11 months	19	15.2		
	1–2 years	24	19.2		
	3–5 years	24	19.2		
	More than 5 years	26	20.8		
Number of employees in company	2–9 people	10	8.0		
	10–50 people	18	14.4		
	51–100 people	13	10.4		
	101–250 people	8	6.4		
	251–500 people	18	14.4		
	501–1,000 people	16	12.8		
	Over 1,000	42	33.6		
**Organization culture**					
Clan				3.44	0.77
Adhocracy				3.01	0.80
Market				3.32	0.73
Hierarchical				3.52	0.76

*N = 124.

The mean of the SIEs age was 35 years old. Slightly more respondents were female (58.4%). The majority of SIEs had a BA, MA, or Ph.D. (88.8%). Stay duration in the host country varied. The majority of SIEs stayed in their host country for more than 5 years (43.2%), 1–2 years (20.0%), or 3–5 years (16.8%). Approximately half of the respondents (52.8%) moved abroad alone, and approximately one quarter moved with a spouse (24.0%) or their whole family (22.4%).

The work-in-company duration was similarly distributed. It varied between 15.2 and 25.6% of SIEs working up to 6 months and 20.8% working for a period of more than 5 years. The size of the companies that they worked for varied from organizations with 2–9 people (8.0%) to companies with over 1,000 employees (33.6%).

### Results of Correlation and Regression Analysis

Correlation and multiple linear regression analyses were conducted to examine the relationship between adjustment and potential predictors (i.e., organizational culture and controlling variables such as individual and work-related predictors). Table 2 summarizes the correlation analysis results. As the results illustrate, Clan and Adhocracy cultures and job factors are positively and significantly correlated with work adjustment. The SIEs with higher scores on indicated variables tend to be more adjusted to work. Meanwhile, Clan culture and controlling variables such as job factors and stay duration positively and significantly correlated with non-work adjustment.

**TABLE 2:** Correlation analysis.

	1	2	3	4	5	6	7	8	9	10	11	12	13	14
1. Work Adj.		0.664**	0.123	–0.028	0.021	0.156	0.003	0.546**	0.201*	0.103	0.403**	0.226*	0.012	0.160
2. Non-work Adj.			0.078	–0.151	0.028	0.225*	–0.006	0.353**	0.123	0.094	0.299**	0.053	–0.122	0.164
3. Age				–0.165	0.048	0.419**	0.422**	0.128	0.554**	0.033	–0.141	–0.047	–0.031	0.000
4. Gender					–0.112	–0.007	–0.164	0.080	–0.096	–0.056	–0.015	0.138	0.182*	0.022
5. Education						0.058	–0.119	0.063	0.073	0.210*	–0.096	–0.113	–0.014	0.148
6. Stay duration							0.179*	0.110	0.721**	0.033	0.029	0.049	0.071	0.150
7. Expatriate moved with (if any)								–0.030	0.231**	–0.001	0.029	–0.123	–0.054	–0.032
8. Job factors									0.271**	0.016	0.386**	0.364**	0.079	0.148
9. Work duration										0.024	–0.022	0.067	–0.011	–0.004
10. Number of employees											−0.200*	−0.267**	–0.090	0.267**
11. Clan												0.575**	0.022	0.212*
12. Adhocracy													0.410**	0.030
13. Market														0.040
14. Hierarchical														

**Correlation is significant at the 0.01 level (2-tailed).

*Correlation is significant at the 0.05 level (2-tailed).

Multiple linear regressions were run to predict the degree of expatriate work and non-work adjustment abroad in relation to organizational culture and individual and work-related variables (see Table 3). Multiple regression models with variables related to (1) individual, (2) individual and work, and (3) individual, work, and organizational culture were produced. The multiple regression models with all 12 predictors (see Step 3) to the work and non-work adjustment produced the following results, respectively: *F*(12, 110) = 8.420, *p* < 0.001, R Square = 0.479 and *F*(12, 110) = 5.671, *p* < 0.001, R Square = 0.382. As Table 3 shows, job factors (*b* = 0.599, *p* < 0.001) and clan organizational culture (*b* = 0.338, *p* < 0.01) have a significantly positive impact on work adjustment. These two variables [respectively, job factors (*b* = 0.480, *p* < 0.001) and clan culture (*b* = 0.322, *p* < 0.05)] also positively influence non-work adjustment. This indicates that SIEs with higher scores on these scales were expected to have a better adjustment in work and non-work adjustment, which corresponds to H1. Furthermore, the duration of stay in the host country (*b* = 0.173, *p* < 0.05) also has a significantly positive impact on non-work adjustment. All other individual-related variables and work-related variables, such as work duration in the company and the number of employees in the company, did not contribute to the multiple regression model on work adjustment. The same can be said for all the other organizational culture types.

**TABLE 3:** Multiple linear regression predicting the degree of expatriates’ adjustment abroad.

Predictors	Work adjustment			Non-work adjustment		
	Step 1	Step 2	Step 3	Step 1	Step 2	Step 3
(Constant)	3.609***	1.461*	0.513	3.781***	2.057***	1.907**
**Step 1**—**Individual related variables**						
Age	0.009	0.004	0.008	0.006	0.006	0.010
Gender	–0.033	–0.133	–0.088	–0.220	−0.318*	–0.230
Education	–0.026	–0.215	–0.185	0.003	–0.142	–0.143
Stay duration in host country	0.086	0.108	0.048	0.131*	0.218**	0.173*
Expatriate moved with (if any)	–0.126	–0.053	–0.099	–0.113	–0.063	–0.126
**Step 2—Work related variables**						
Job factors		0.774***	0.599***		0.576***	0.480***
Work in company duration		–0.080	–0.016		−0.170*	–0.121
Number of employees in company		0.027	0.042		0.032	0.037
**Step 3—Organization culture**						
Clan			0.338**			0.322*
Adhocracy			–0.085			–0.217
Market			0.102			–0.018
Hierarchical			0.022			–0.019

*F*	0.961	10.431***	8.420***	2.083	6.956***	5.671***
*R Square*	0.039	0.423	0.479	0.082	0.328	0.382
*Change in R Square*	0.039	0.384	0.056	0.082	0.246	0.054

Significant at *p < 0.05, **p < 0.01, ***p < 0.001.

Our study revealed a significant positive correlation between Adhocracy culture and work adjustment (*r* = 0.226, *p* < 0.05); however, the regression analysis did not indicate the impact of Adhocracy culture on work adjustment. Therefore, H2a is not supported. These findings correspond in part to Lund’s (2003) study, where a positive impact on work adjustment was discovered while studying local employees. No statistically significant relation was found between Adhocracy culture and general non-work adjustment, not supporting H2b.

Our assumptions that less people-oriented and not flexible Marketing and Hierarchical organizational cultures negatively influence work adjustment and non-work adjustment and our H3 and H4 were not supported. No statistically significant relationship was found between these constructs. Therefore, it could be explained that Marketing and Hierarchical cultures do not influence adjustment and fit organization of expatriates neither helping nor harming this process.

## Discussion

This study examined the influence that organizational culture type has on self-initiated expatriate adjustment. The empirical analysis attempted to assess the extent to which the work and non-work adjustment of SIEs, in particular, could be explained by organizational culture type. Several interesting results emerged from the analysis.

Hypothesis 1 suggests that organizational culture types that promote close relationships, communication, and teamwork positively correlate with work and non-work adjustment. Results showed a positive impact of Clan culture on work and non-work-related adjustment and supported H1. Clan culture thus appears to offer the best fit between the organization and the person, corresponding with the P-O fit theory. As Pinto et al. (2011) noted, frequent social interactions are required to establish relationships in an organization, and expatriates who do not have local networks have trouble meeting these conditions. Clan culture is like an extended family and characterized by teamwork, consensus, and participation, which makes it easier for expatriates to make social connections. In this type of work environment, SIEs can establish friendships, which is important for foreigners living in a host country. These insights align with Haslberger et al.’s (2014) belief that interaction with people is important for both work and non-work adjustment. Moreover, we note that the work-related adjustment of SIEs could be explored in relation to new local employees. Our findings correspond with Lund’s (2003) results, showing that family-relation-based culture helps new employees adjust inside an organization.

The results revealed that individual-related control variables, such as age, gender, and education, do not correlate with work and non-work adjustment. These results do not support the findings of Pinto et al. (2011), who explored assigned expatriates; however, with respect to gender issues, Selmer and Leung (2003) also did not find a relationship between gender difference and non-work adjustment, though they did find that female expatriates had higher levels of work adjustment than males. In relation to gender, more contradicting results could be found. Pinto et al. (2011) found that female expatriates have more non-work adjustment difficulties than male expatriates, but this does not correspond with the results of our study or the work of Selmer and Leung (2003). This difference may stem from the fact that SIEs differ from AEs in that they are more self-oriented. Also, more females recently decided to take international assignments on their own, so these gender issues could become less prevalent in the future. Nevertheless, these insights need deeper support and need to be explored in more detail in future studies.

It was also indicated that stay duration has a positive impact on non-work adjustment. This finding was unsurprising, as time spent abroad allows individuals to become more familiar with a country’s culture and environment.

After analyzing work-related control variables, years spent in an organization did not predict work adjustment, which aligns with the findings of Stroh et al. (1994); however, interestingly enough, time spent in an organization was negatively associated with non-work adjustment, which calls for additional analysis that explores this issue in more detail. Finally, job factors related to role clarity and freedom at work positively contribute to work and non-work adjustment and confirm the previous findings of Black (1988) and Stroh et al. (1994).

Overall, our results highlight that Clan culture type and job factors affect the work and general non-work adjustment of self-initiated expatriates.

### Theoretical Contributions

First, this study expands our understanding of SIE adjustment. It contributes to the scarce SIE management literature by investigating the understudied topic of the role that organizational culture typology plays in the adjustment process. Second, this study in relation to O’Reilly et al. (1991) research, who revealed the importance between an individual’s preference for a particular culture features in P-O fit, adds primary insights that particular organizational culture such as Clan culture that is based on friendly and supportive relations and values fits SIE’s values foster SIEs adjustment and fit to the organization.

### Implications for Managerial Practice

This study is important from a practical point of view. Retention of employees is a serious concern for organizations. If employees do not adjust, they leave the organization, and then the organization spends extra time and money on new employee hiring, training, and adjustment. As the flow of SIEs is growing globally, their adjustment issues are becoming very important. Moreover, foreign employees are becoming a norm in both international and local organizations. As local organizations do not have deep experience and have developed procedures to employ foreign employees, they could face more problems with SIE adjustment and retention. To successfully adjust in local organizations, SIEs need support from inside the organization; however, not much research has been done exploring how human resource groups in local organizations train local managers for an influx of foreign employees. Based on our results, we could suggest that companies hiring SIEs should develop an organizational culture based on trust, flexibility, and high sociability. Its key features should be friendly relationships, open communication, and informality between group members. This will bring satisfaction, loyalty, and commitment to the work of employees and will increase their retention. Furthermore, SIEs influence the organizational culture of their companies, and local employees also need support and training to adapt to working within international teams to understand cultural, communication, and work-related differences. Therefore, the SIE adjustment issues should be studied more widely and expanded to all aspects of organizational existence.

## Conclusion

The main conclusion of this study is that organizational culture type could foster the adjustment of self-initiated expatriates self-initiated at work and in their host country. The study’s findings encourage the use of the P-O fit theory to explain the work adjustment of SIEs. Following the P-O fit theory’s approach, we determined that the best fitting culture (i.e., the one that corresponds with SIE needs and values and helps them adjust) is Clan culture. This type of culture has a positive relation to the work and non-work adjustment of self-initiated expatriates. Moreover, the results demonstrate that organizational culture type rather than time spent in an organization influences expatriate work adjustment, which highlights the importance of this study.

### Limitations and Future Research Directions

The first limitation of this research is the use of a cross-sectional design, as all variables were collected through the same questionnaire. The second limitation concerns the use of self-reported data, which may be under the influence of common method variance. To prevent this, however, we shortened the questions and the questionnaire as much as we could, but we still received a low percentage of responses. Actually, larger international samples are difficult to access and, as Shaffer et al. (2006) have noted, the response rate for expatriates is low, averaging only 15%. Therefore, this study design was considered adequate to address the research questions. Another limitation is that the survey was limited to those with access to the Internet and who self-selected to participate; however, this kind of mean is adequate, and the majority of expatriates use the Internet for their communication, especially in their home country. Consequently, we suggest that future studies use multiple data collecting methods. Our research was also limited with respect to the different periods of time that SIEs spent in their host countries. Larger samples that include respondents who spent a similar amount of time in their host country or samples that focus on fixed short-term stays could be used in future studies. This study neglected the culture of the host country, as the research focused on a world sample. Studies that center nationals from a single country who expatriated to different countries or expatriates from different home countries who reside in the same host country could be pursued in the future. We also did not test culture novelty, spouse or family adjustment, previous international experience, and host language skills in the current study, which should be explored in future studies.

Finally, a theoretical limitation relates to the P-O fit theory. It is limited when applied to organizational levels and work adjustment and thus could be expanded by person-environment fit theory if researching the non-work adjustment of expatriates. Moreover, other typologies of organizational culture could be analyzed in future studies.

## Data Availability Statement

The raw data supporting the conclusions of this article will be made available by the authors, without undue reservation.

## Author Contributions

All authors contributed to the design and implementation of the research, the analysis of the results, and the writing of the manuscript.

## Conflict of Interest

The authors declare that the research was conducted in the absence of any commercial or financial relationships that could be construed as a potential conflict of interest.

## Publisher’s Note

All claims expressed in this article are solely those of the authors and do not necessarily represent those of their affiliated organizations, or those of the publisher, the editors and the reviewers. Any product that may be evaluated in this article, or claim that may be made by its manufacturer, is not guaranteed or endorsed by the publisher.
